# 

**Published:** 1884-07

**Authors:** 


SUBSCRIPTION PRICE, $3.00 PER YEAR.

Whole Number, 295. JULY, 1884. Vol. XLIX, Number 1.

LIBRARY
INDEXED
S. G. O.

### THE CHICAGO MEDICAL JOURNAL AND EXAMINER

EDITORS:

JAMES NEVINS HYDE.

W. W. JAGGARD. HAROLD N. MOYER.

CHICAGO:

PUBLISHED BY THE CHICAGO MEDICAL PRESS ASSOCIATION,

193 WEST MADISON STREET.

COWDREY, CLARK & CO., PRINTERS, 163 AND 165 DEARBORN STREET, CHICAGO.

